# The spider family Filistatidae (Araneae) in Iran

**DOI:** 10.3897/zookeys.516.10146

**Published:** 2015-08-10

**Authors:** Yuri M. Marusik, Alireza Zamani

**Affiliations:** 1 Institute for Biological Problems of the North RAS, Portovaya Str. 18, Magadan 685000, Russia; 2 Department of Zoology & Entomology, University of the Free State, Bloemfontein 9300, South Africa; 3 Far Eastern Federal University, Sukhanova 8, Vladivostok 690950, Russia; 4 Department of Animal Biology, School of Biology and Centre of Excellence in Phylogeny of Living Organisms in Iran, College of Sciences, University of Tehran, Tehran, Iran; 5 Pars Plateau Zoologists Group, Iran

**Keywords:** Fauna, Aranei, Near East, new species, *Filistata*, *Sahastata*, *Zaitunia*

## Abstract

All species of Filistatidae occurring in Iran are surveyed. *Zaitunia akhanii* **sp. n.** is described on the basis of female specimens collected in Tehran province, and the previously unknown male of *Sahastata sinuspersica* Marusik, Zamani & Mirshamsi, 2014 is described for the first time. Also, the endogynes of the holotypes of *Zaitunia alexandri* Brignoli, 1982, *Zaitunia medica* Brignoli, 1982 and *Zaitunia persica* Brignoli, 1982 are illustrated. Including these results, the number of Iranian species of Filistatidae is increased to seven, which indicates the highest species-richness of this family in the Western Palaearctic.

## Introduction

Filistatidae is a relatively small, globally-distributed family currently comprised of 119 extant species within 18 genera (World Spider Catalog 2015). The family has never been revised at the global scale. Filistatids are relatively well-studied in the West Palaearctic due to Brignoli (1982), who surveyed all species known from the Iberian Peninsula to Iran. In that paper, Brignoli described three new species of *Zaitunia* Lehtinen, 1967 and reported *Filistata insidiatrix* (Forskal, 1775) from Iran for the first time. The taxonomy of Filistatidae of Iran has been dealt with in three publications only (Zamani et al. 2015). The second paper dealing with Iranian Filistatidae was published by Marusik and Zonstein (2014), where they surveyed the Middle East *Filistata* Latreille, 1810, described a new species from Azerbaijan, and provided taxonomic and faunistic data regarding *Filistata insidiatrix* in Iran. The third paper was published by Marusik et al. (2014), in which the genus *Sahastata* Benoit, 1968 was recorded from Iran for the first time, and a new species, *Sahastata sinuspersica* Marusik, Zamani & Mirshamsi, 2014, was described on the basis of female specimens collected in southern Iran. In addition, four faunistic papers have been published that provided additional information regarding the distribution of *Filistata insidiatrix* in Iran (Ghahari and Marusik 2009, Ghahari and Tabari 2012, Tabrizi et al. 2014, Zamani 2015) and one recent publication provided the first Iranian record of *Filistata lehtineni* Marusik & Zonstein, 2014 (Moradi et al. in press). In this study, one new species and the male of *Sahastata sinuspersica* are described and all taxonomic and faunistic data published regarding this family in Iran are provided.

## Materials and methods

Specimens were photographed using an Olympus Camedia E-520 camera attached to an Olympus SZX16 stereomicroscope or to the eye-piece of an Olympus BH-2 transmission microscope. Digital images were prepared using “CombineZP” image stacking software (http://www.hadleyweb.pwp.blueyonder.co.uk/). Illustrations of internal genitalia were made after clearing in 10% KOH aqueous solution and exposure for a few minutes in an alcohol/water solution of Chlorazol Black. Lengths of leg segments were measured on the dorsal side. Measurements of palp and legs are listed as: total length [femur, patella, tibia, metatarsus, tarsus]. Description of the palp refers to the left one. All measurements are given in millimeters.

### Depositories

MCSN Museo Civico di Storia Naturale di Verona.

SMF Senckenberg Museum, Frankfurt am Main.

ZMMU Zoological Museum of the Moscow State University.

ZMUT Zoological Museum of University of Tehran.

## Taxonomy

### Genus Filistata

Taxon classificationAnimaliaAraneaeFilistatidae

Latreille, 1810

#### Type species.

*Filistata testacea* Latreille, 1810 (considered a junior synonym of *Filistata insidiatrix*).

*Filistata* is a genus of medium to large-sized Filistatinae spiders with 19 valid species mainly distributed from Mediterranean to Turkmenistan. Members of this genus can be diagnosed by the long and cylindrical palpal tibia of males, well-developed thoracic fovea, long and subhorizontal clypeus, oval sternum and longer than wide labium (Zonstein et al. 2013).

### Filistata insidiatrix

Taxon classificationAnimaliaAraneaeFilistatidae

(Forskål, 1775)

Figs 1c–d , 7

Filistata insidiatrix : Brignoli 1982: 68, f. 1–5 (♂♀); Ghahari and Marusik 2009: 4 (distribution record); Ghahari and Tabari 2012: 139 (distribution record); Marusik and Zonstein 2014: 200, f. 1–3, 7–8, 11–12, 15–16, 19–22, 25–27 (♂♀); Tabrizi et al. 2014: 30 (distribution record); Zamani 2015: 12 (distribution record).
Filistata insidiatrix For the complete list of taxonomic references see World Spider Catalog (2015).

#### Diagnosis.

**Figure 1.**
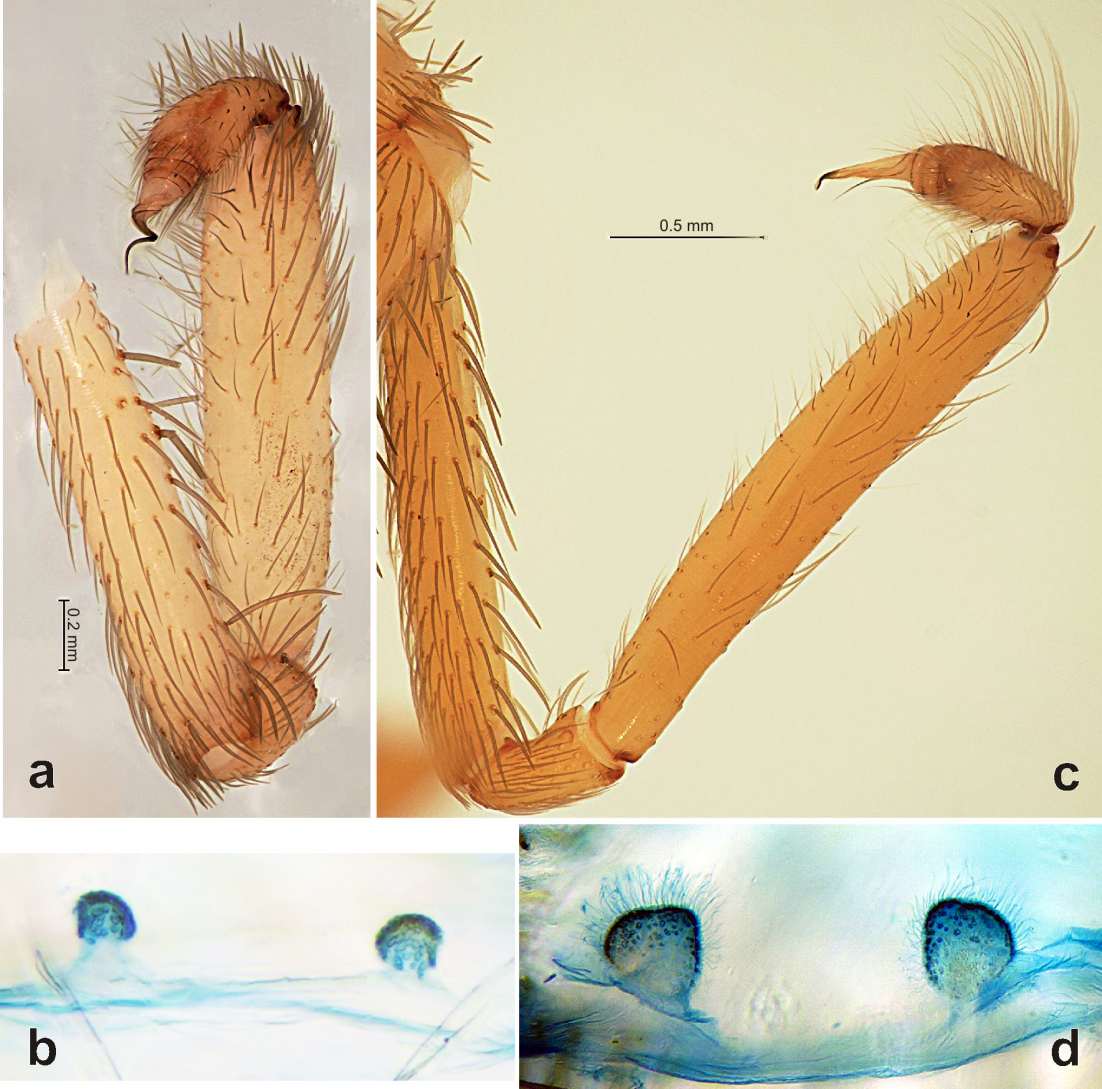
Copulatory organs of *Filistata lehtineni* (**a–b**) and *Filistata insidiatrix* (**c–d**). **a, c** male palp, retrolateral **b, d** endogyne, dorsal. After Marusik and Zonstein (2014).

This species differs from *Filistata lehtineni* by larger size, longer male palp, and larger receptacles (*cf.* Fig. 1a–d).

#### Description.

Well-described by Brignoli (1982).

#### Records in Iran.

Chahar Mahal & Bakhtiary, Fars, Golestan, Isfahan, Kordestan, Mazandaran, Razavi Khorasan, Tehran.

#### Global distribution.

This species has the widest range within the entire family. It is known from the Iberian Peninsula to Turkmenistan and northeastern Iran (Marusik and Zonstein 2014). The record of this species from Razavi Khorasan is the easternmost in its range.

### Filistata lehtineni

Taxon classificationAnimaliaAraneaeFilistatidae

Marusik & Zonstein, 2014

Figs 1a–b , 7

Filistata lehtineni Marusik and Zonstein 2014: 202, f. 4–6, 9–10, 13–14, 17–18, 23–24, 28–30 (♂♀); *Filistata lehtineni*: Moradi et al. in press (♂).

#### Diagnosis.

This species differs from *Filistata insidiatrix* by smaller size, shorter and thicker male palp with screw-shaped embolus, and smaller receptacles (*cf.* Fig. 1c–d).

#### Description.

Both sexes of this species are described in detail in Marusik and Zonstein (2014).

#### Record in Iran.

Zanjan.

#### Global distribution.

Known only from southern Azerbaijan and northwestern Iran.

### Genus Sahastata

Taxon classificationAnimaliaAraneaeFilistatidae

Benoit, 1968

#### Type species.

*Filistata nigra* Simon, 1897.

*Sahastata* is a small genus of large-sized Filistatinae spiders with four described species distributed from the Mediterranean to India. Members of *Sahastata* differ distinctly from other genera of Filistatinae by the shape of the calamistrum (two-three rows, not placed in a crest), presence of a dense ventral scopula on the femora I and II of females (Benoit 1968), dense hairs on female sternum and labium and several small spines on the femora I and II of males. So far, *Sahastata* males are known only by one species from India, *Sahastata ashapuriae* Patel, 1978. Unfortunately, the description of this species lacks several important characters, such as female internal genitalia and cribellum and male legs spination (*cf.* Patel 1978), which are critical for the genus recognition and the separation of species.

### Sahastata sinuspersica

Taxon classificationAnimaliaAraneaeFilistatidae

Marusik, Zamani & Mirshamsi, 2014

**Figure 2.**
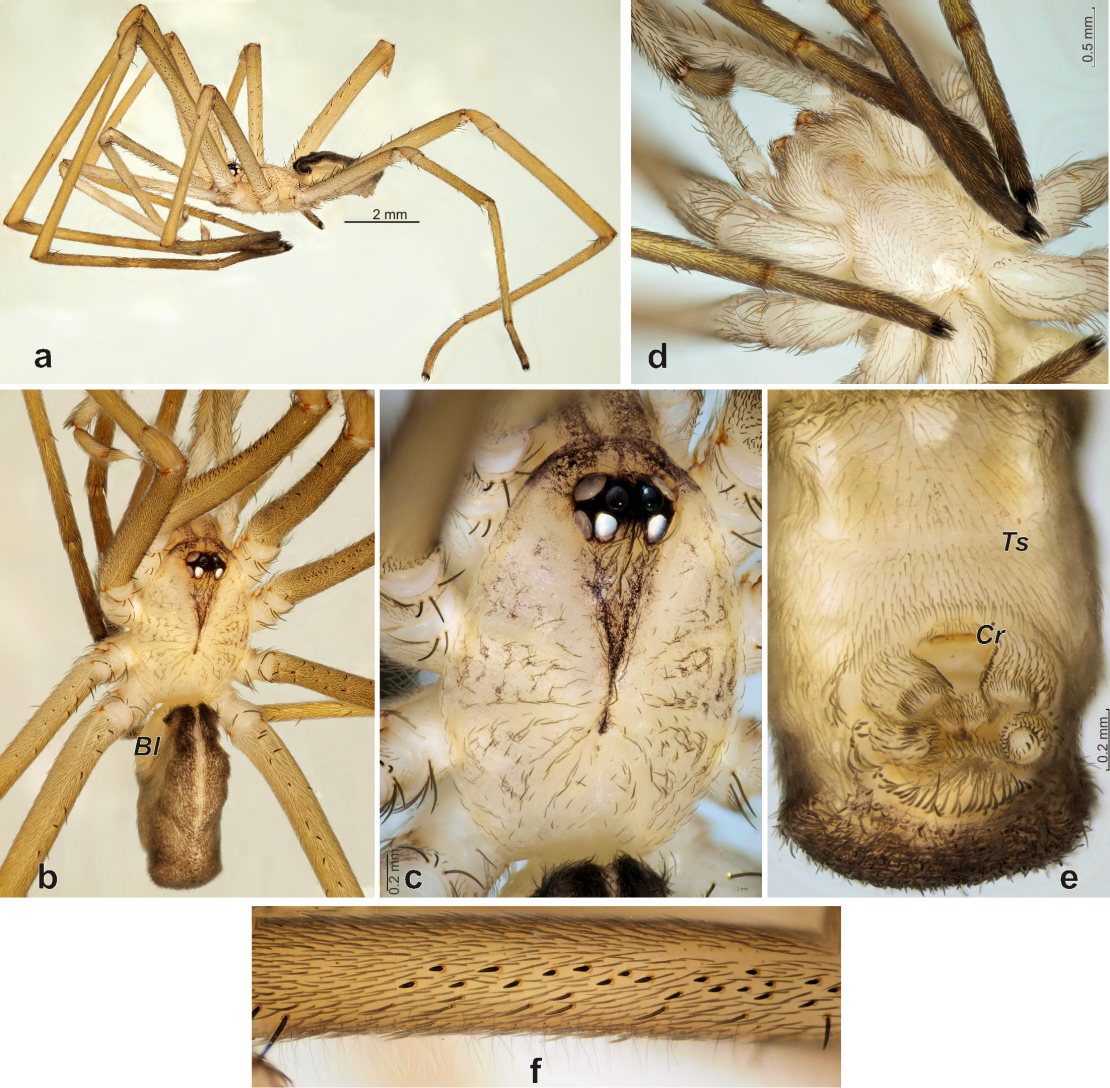
Somatic characters of *Sahastata sinuspersica*, male. **a, b** habitus, lateral and dorsal **c** carapace, dorsal **d** prosoma, ventral **e** abdomen, ventral **f** part of femur I showing spination, prolateral. Abbreviations: *Bl* book lung, *Cr* cribellum, *Ts* tracheal spiracle.

**Figure 3.**
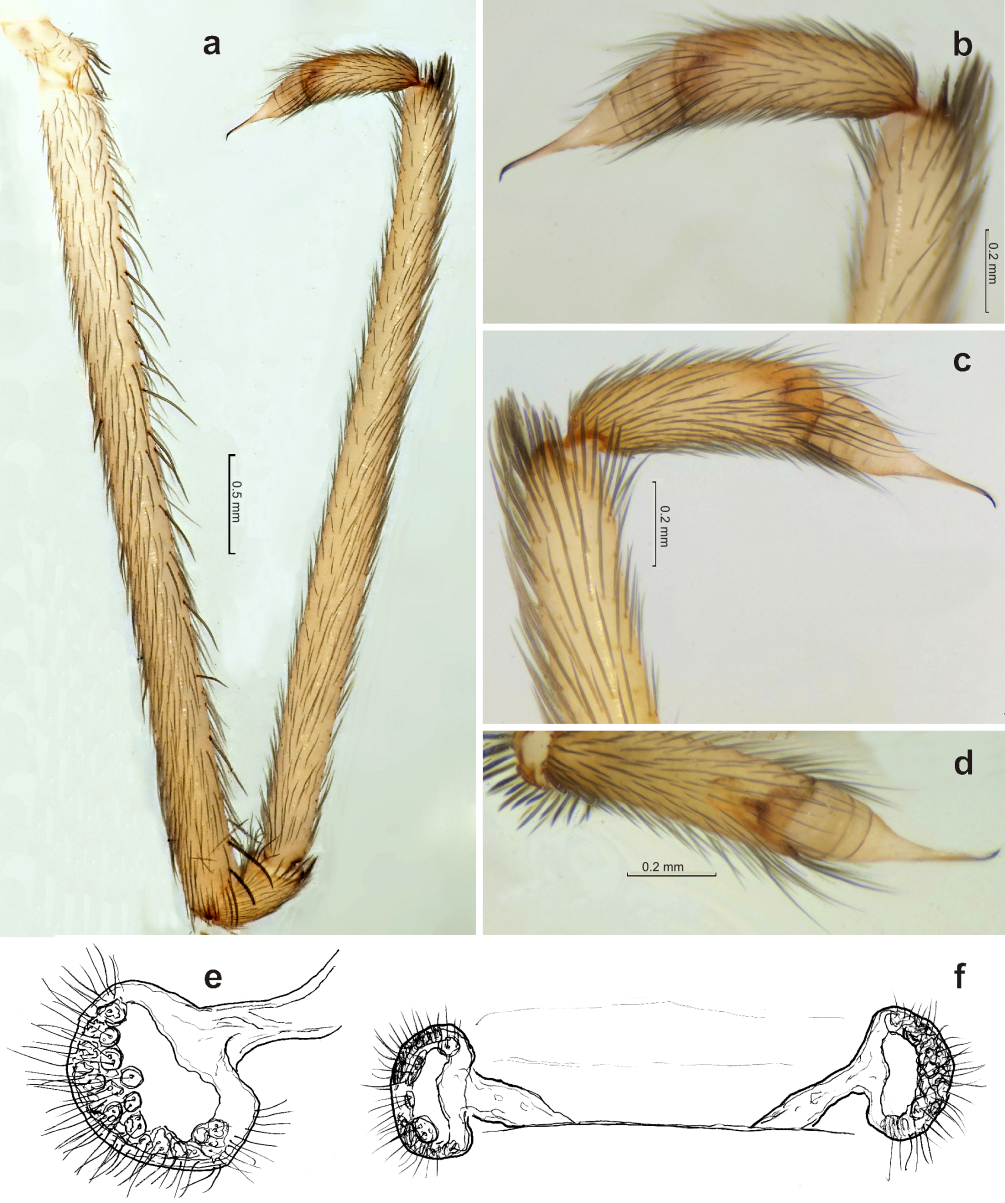
Copulatory organs of *Sahastata sinuspersica*. **a** whole male palp, retrolateral **b–d** terminal part of the male palp, retrolateral, prolateral and from above **e** receptacle, dorsal **f** endogyne, dorsal.

Figs 2 , 3 , 6b–c , 7

Sahastata sinuspersica Marusik et al. 2014: 9, f. 22–29, 34–40 (♀).

#### Material examined.

IRAN: 1♂ 1♀ (SMF), *Hormozgan Province*: Hormuz Island, 27°04'N, 56°28'E, January 2015 (A. Zamani).

#### Diagnosis.

Females of *Sahastata sinuspersica* can be distinguished from the other female congeners by having one pair of receptacle heads connected to the epigastric furrow by a pair of ducts, while *Sahastata nigra* Simon, 1897 present two pairs of spermathecae heads (Benoit 1968: fig. 4) and *Sahastata sabaea* Brignoli, 1982 has the duct connected to the bursa copulatrix (Brignoli 1982: fig. 18) (Fig. 3e–f). The male differs from all known filistatids except for *Filistata puta* O.Pickard-Cambridge, 1876 (*sensu* Wunderlich 1995) by having numerous small spines on femora I and II. Males of *Sahastata sinuspersica* and *Filistata puta* both have relatively long palps but differ by the shape of the bulb: conical and tapering in *Sahastata sinuspersica* and with round tegular part in *Filistata puta* (Wunderlich 1995: figs 2–4). The bulb of *Sahastata sinuspersica* is very similar to that in *Sahastata ashapuriae*. Although the latter species is poorly-described and illustrated, it can be easily distinguished from *Sahastata sinuspersica* by having palps longer than leg I (palp twice shorter than leg I in Iranian species).

#### Description.

Male. Total length 4.85. Carapace 2.32 long, 1.75 wide, 0.5 high, flat, light-colored, with V-shaped brown median spot reaching clypeus and poorly distinct radial stripes, covered with short adpressed dark hairs, postocular area with few strong erected hairs (Fig. 2b–c). Eye tubercle moderately elevated, brownish-black. Chelicerae with median brown bands (Fig. 2b–c). Sternum uniformly light-colored (Fig. 2d), hairs covering sternum not as dense as in female (Marusik et al. 2014: fig. 24). Legs light brownish-yellow, darker than carapace, metatarsi and tarsi darker than other segments due to dense brownish hairs (Fig. 2a, d). Legs very long, first leg four times longer than body (Fig. 2a). All legs with distinct spines, femora I with numerous pro- and retrolateral small spines (Fig. 2b, f), femora II with less dense spination prolaterally. All leg tarsi with pseudosegmentation (cuticular cracks) (Fig. 2a, d). Calamistrum absent. Measurements of palp and legs: Palp 9.66 [4.5, 0.5, 4.03, 0.63], I 19.65 [5.25, 1.05, 5.85, 5.5, 2.0], II 13.75 [3.85, 1.0, 3.65, 3.75, 1.5], III 11.9 [3.25, 0.95, 2.8, 3.5, 1.4], IV 16.65 [4.5, 1.05, 4.3, 4.8, 2.0]. Abdomen brownish, with dark brown anterior part of dorsum and distinct light median stripe. Book lungs (*Bl*) very large (length about 1/3 of abdomen length) (Fig. 2b), tracheal spiracle (*Ts*) wide, located almost on half way from epigastric furrow to cribellum (Fig. 2e). Cribellum (*Cr*) present (Fig. 2e), large, transverse and divided.

Palp as in Fig. 3a–d, very long, two times longer than body, femur as long as femur of leg IV, covered with spines; patella very short, shorter than cymbium; tibia slightly thinner than femur, without spines; cymbium cylindrical, longer than free part of bulb; bulb conical gradually tapering, embolic part not well-separated from tegular part, shorter than tegular part; tip of embolus slightly bent retrolaterally; Spermophor with three coils in retrolateral and two coils in prolateral.

*Female*. Described by Marusik et al. (2014).

#### Habitat.

Specimens were mostly found under stones and in natural crevices on a sandy substrate near the sea.

#### Records in Iran.

Hormozgan.

#### Distribution.

Endemic to southern Iran.

### Genus Zaitunia

Taxon classificationAnimaliaAraneaeFilistatidae

Lehtinen, 1967

#### Type species.

*Filistata schmitzi* Kulczyński, 1911.

*Zaitunia* is a small genus of small to medium-sized Filistatinae spiders with 11 described species distributed from East Mediterranean to Central Asia. They are diagnosable from the similarly-looking *Filistata* by the lack of a thoracic fovea, short and subvertical clypeus, subcircular sternum, as broad as long labium, and by a short and swollen palpal tibia of males (Zonstein 2009, Zonstein et al. 2013).

### Zaitunia akhanii sp. n.

Taxon classificationAnimaliaAraneaeFilistatidae

http://zoobank.org/61D6F60B-59E9-4E87-973A-F46DB5BE979B

Figs 4 , 6a , 7

#### Material examined.

IRAN: Holotype ♀ (SMF) and paratypes 7♀ (ZMMU, ZMUT), *Tehran Province*: Southern macroslopes of Alborz mountains, 35°48'29"N, 51°23'E, July 2014 (A. Zamani).

#### Etymology.

This species is named after Iranian botanist Hossein Akhani (University of Tehran), in recognition of his contributions to the botanical studies of Iran and his numerous environmental activities.

#### Diagnosis.

Females of *Zaitunia akhanii* sp. n. resemble those of *Zaitunia persica* Brignoli, 1982 by having one pair of sinuous tube-like receptacles, but *Zaitunia akhanii* sp. n. has two loops (or curves) while *Zaitunia persica* has four (Brignoli 1982: fig. 14).

#### Description.

**Figure 4.**
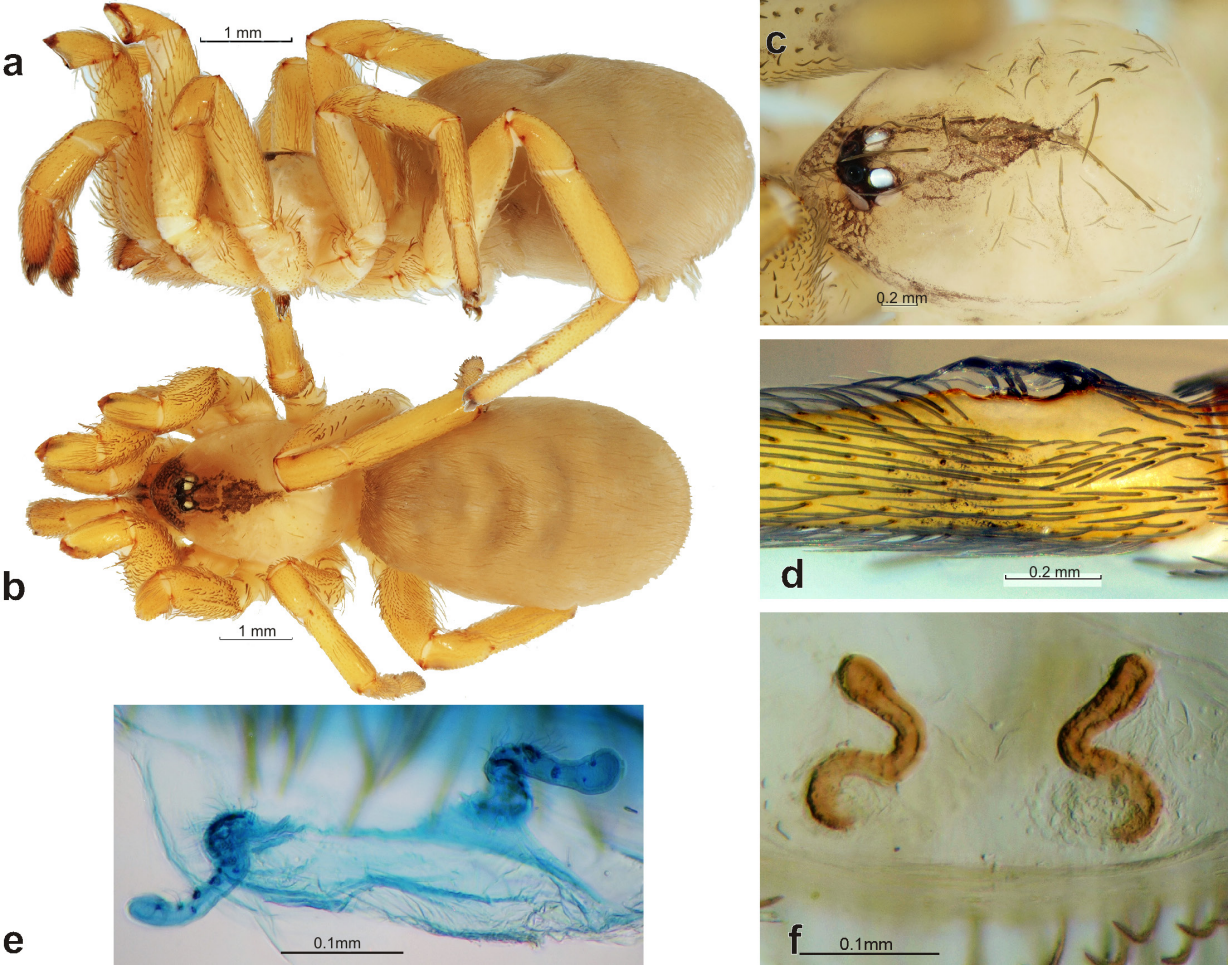
Holotype of *Zaitunia akhanii* sp. n. **a–b** habitus, lateral and dorsal **c** carapace, dorsal **d** calamistrum **e–f** endogyne, anterior and ventral.

Female (paratype). Total length 5.2. Carapace 2.16 long, 1.6 wide. Eye sizes and interdistances: AME 0.09, ALE 0.16, PLE 0.11, PME 0.12, AME-AME 0.03. Light yellowish-colored with distinct pattern on carapace and legs: clypeus whole dark, wide dark median band terminated near fovea. Abdomen uniformly yellowish-gray without darker pattern. Legs with few spines; calamistrum located on low ridge, uniseriate (Fig. 4d). Measurements of palp and legs: Palp 3.28 [1.2, 0.6, 0.68, 0.8], I 9.4 [3.12, 0.8, 2.28, 2.0, 1.2], II 6.28 [1.76, 0.72, 1.48, 1.44, 0.88], III 5.28 [1.52, 0.6, 1.2, 1.2, 0.76], IV 7.12 [2.08, 0.8, 1.68, 1.68, 0.88].

Vulva as in Fig. 4e–f, with one pair of sinuous tube-like receptacles. Receptacles wavy, bent two times, glands not distinct in low magnification but well visible after contrasting coloring (Fig. 4e); glands distributed along whole receptacle and denser in the basal half.

*Male*. Unknown.

#### Variations.

Total length 4.8–7.2. Pale specimens may have light clypeus.

#### Habitat.

Specimens were found in large, dusty cribellate webs made around human dwellings.

#### Distribution.

Known only from the type locality in Tehran.

### Zaitunia alexandri

Taxon classificationAnimaliaAraneaeFilistatidae

Brignoli, 1982

Figs 5b , 7

Zaitunia alexandri Brignoli 1982: 74, f. 15 (♀).

#### Type.

IRAN: holotype ♀ (MCSN), *Fars Province*: Kuhenjan, 27 May 1976 (S. Zerunian).

#### Diagnosis.

This species differs from other Iranian congeners by the shape of the sac-like receptacles, slightly longer than wide.

#### Description.

Well-described by Brignoli (1982).

**Figure 5.**
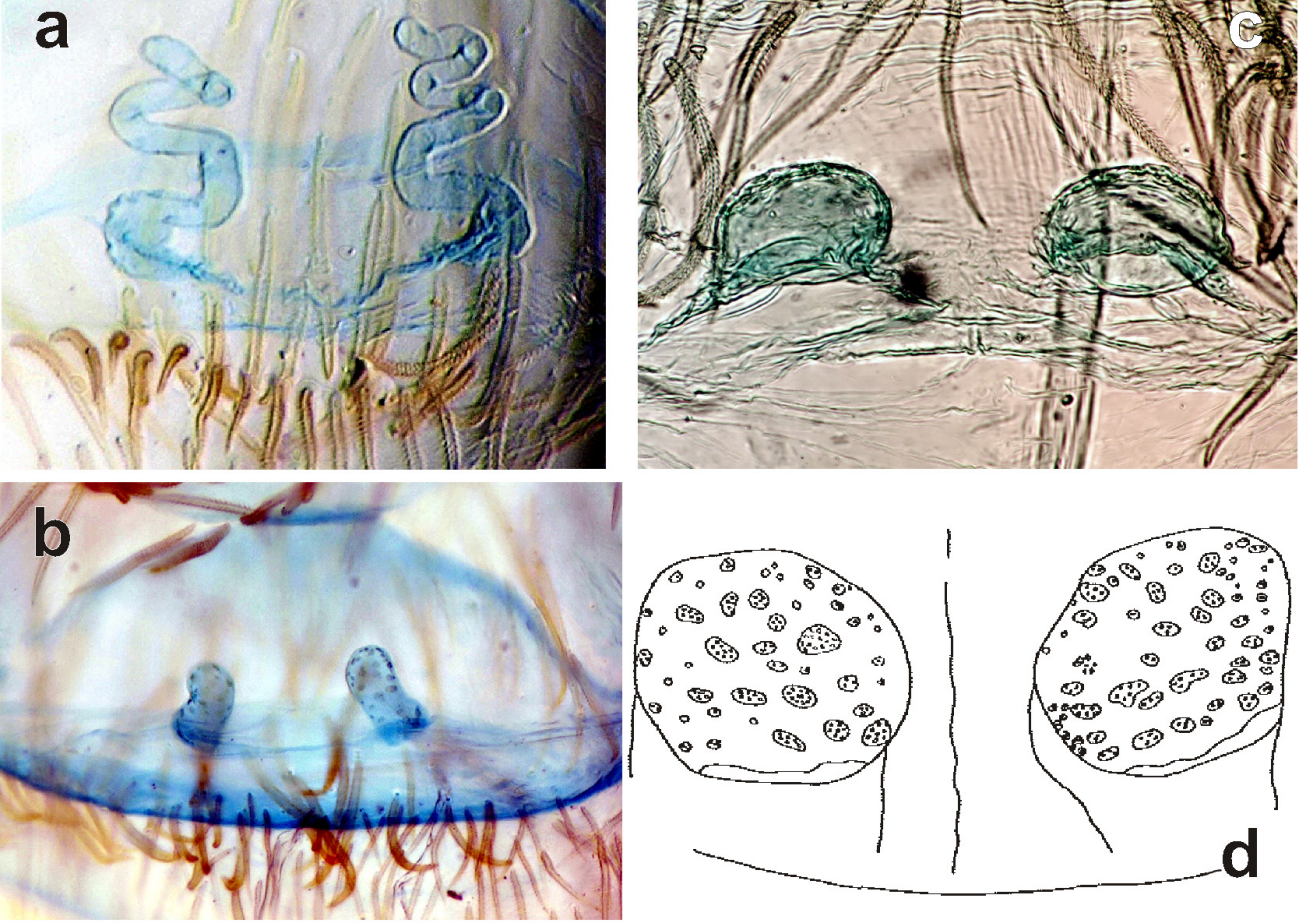
Dorsal view of endogynes of the holotypes of *Zaitunia persica* (**a**), *Zaitunia alexandri* (**b**) and *Zaitunia medica* (**c–d**). 6d after Brignoli (1982).

#### Record in Iran.

Fars.

#### Distribution.

Endemic to southern Iran.

### Zaitunia medica

Taxon classificationAnimaliaAraneaeFilistatidae

Brignoli, 1982

Figs 5c–d , 7

Zaitunia medica Brignoli 1982: 72, f. 16 (♀).

#### Types.

IRAN: holotype ♀ and paratype ♀ (MCSN), *Isfahan Province*: Laybid, 2100 m, 7 July 1975 (P. Brignoli & M. Di Rao).

#### Diagnosis.

This species differs from other Iranian congeners by the shape of sac-like receptacles, which are wider than long.

#### Description.

Well-described by Brignoli (1982).

#### Record in Iran.

Isfahan.

#### Distribution.

Endemic to central Iran.

### Zaitunia persica

Taxon classificationAnimaliaAraneaeFilistatidae

Brignoli, 1982

Figs 5a , 7

Zaitunia persica Brignoli 1982: 70, f. 13–14 (♀).

#### Types.

IRAN: holotype ♀ and paratype ♀ (MCSN), *Fars Province*: Dehbid, 2100m, 24 May 1976 (P. Brignoli).

#### Diagnosis.

This species differs from other Iranian congeners by very long, tube-like receptacles curved four times.

#### Description.

Well-described by Brignoli (1982).

#### Record in Iran.

Fars.

#### Distribution.

Endemic to southern Iran.

**Figure 6.**
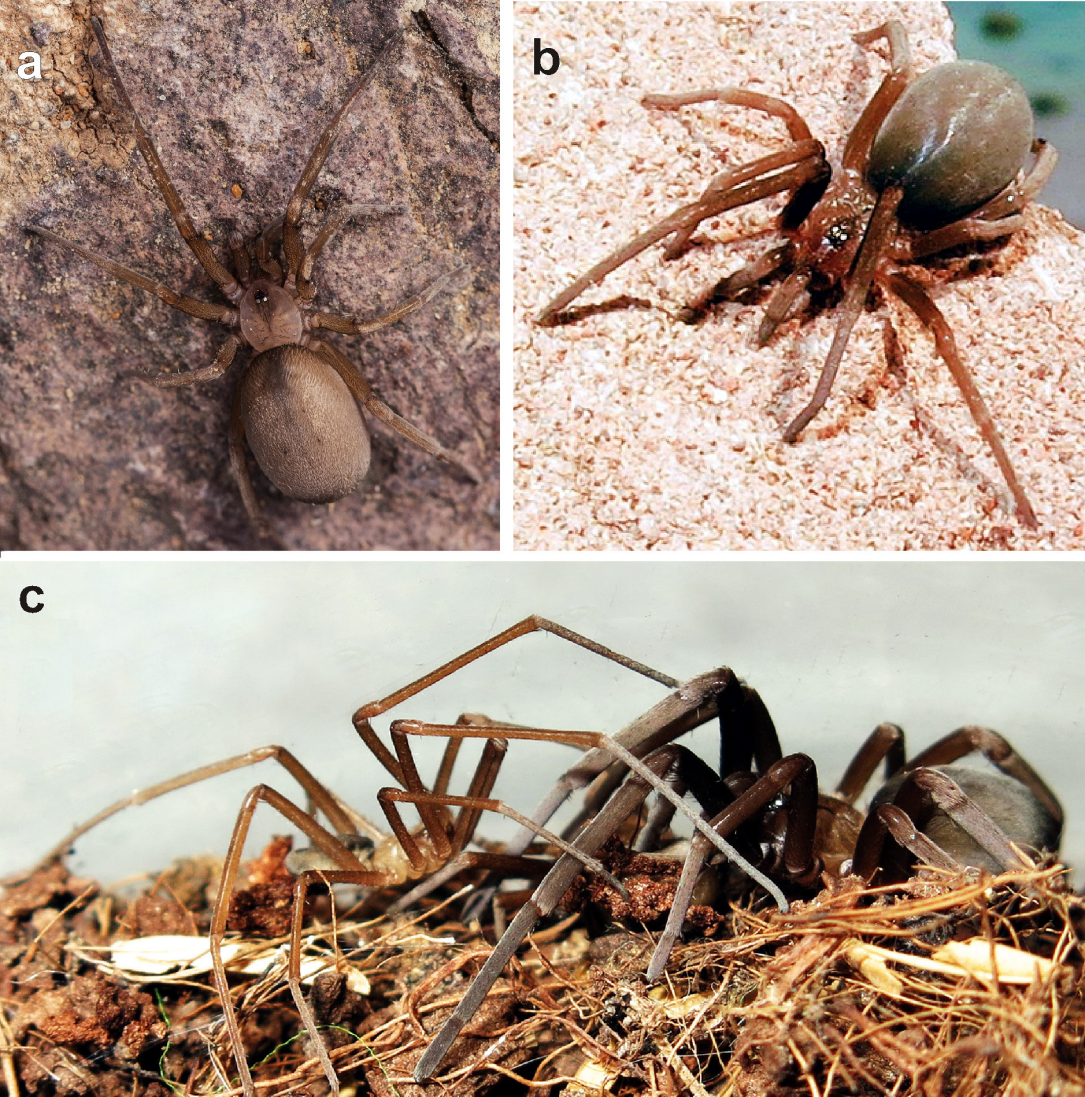
Live specimens of *Zaitunia akhanii* sp. n. (**a**), and *Sahastata sinuspersica* (**b–c**). **a–b** female, dorsal **c** male (left) and female (right) prior to copulation, on artificial surface. Photographs by A. Mohajeran (**a**) and A. Zamani (**b–c**).

**Figure 7.**
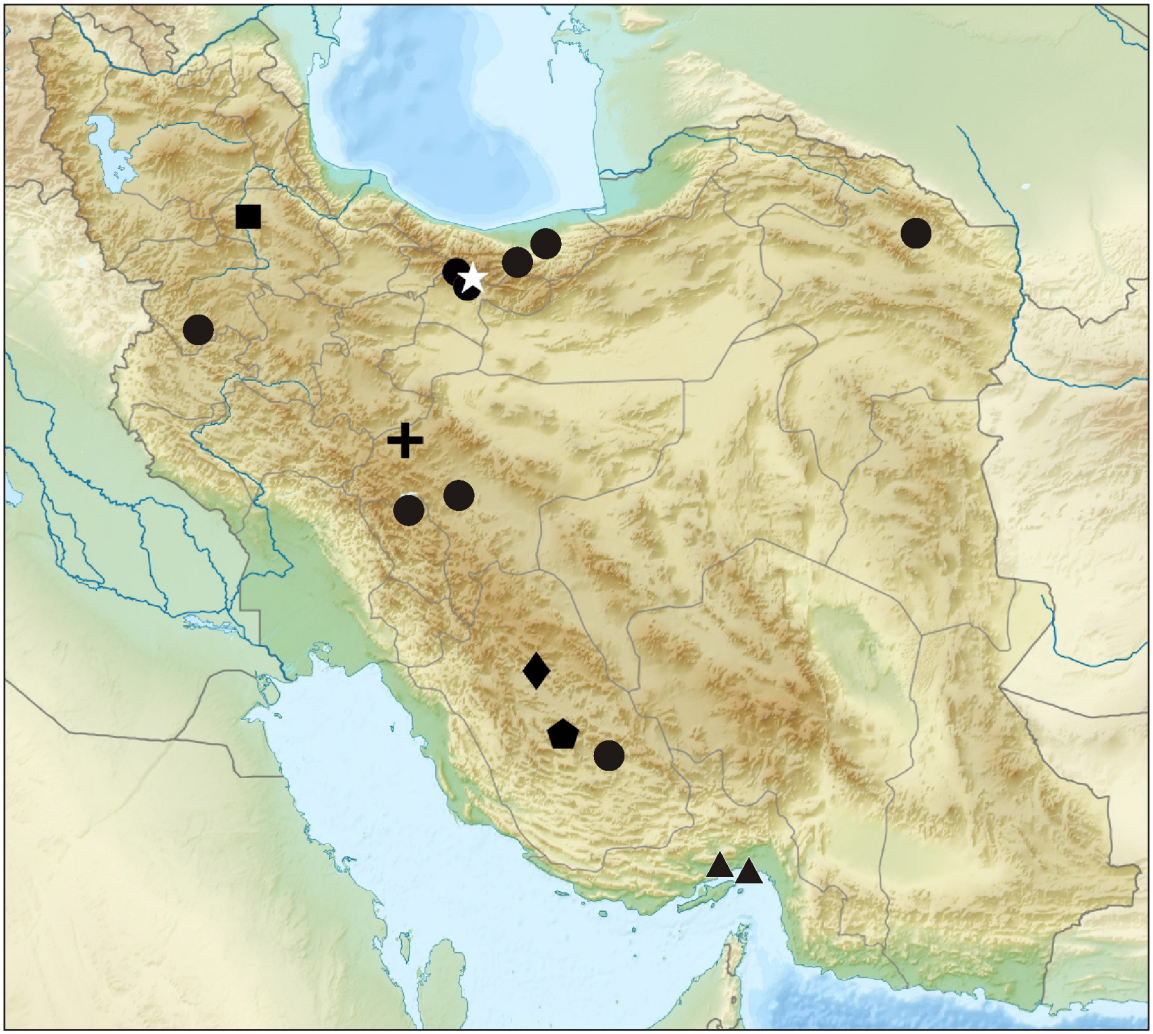
Distribution records of filistatids in Iran: *Filistata insidiatrix* (circle), *Filistata lehtineni* (square), *Sahastata sinuspersica* (triangle), *Zaitunia akhanii* sp. n. (star), *Zaitunia alexandri* (pentagon), *Zaitunia medica* (cross) and *Zaitunia persica* (diamond).

## Conclusions

Although some other species of *Zaitunia* described from nearby countries have not been properly described and their genitalia have never been illustrated, and female filistatids are known to be morphologically variable to some degrees, an ongoing revision of this genus (Zonstein and Marusik, unpublished) and the examination of more than 20 species, including types of all central Asian species (which all have very limited distributions) confirm that *Zaitunia akhanii* sp. n. is a separate, undescribed species. The results of this study show that there are seven species in three genera of Filistatidae known from Iran, of which five are endemic and one is sub-endemic. This is the highest species-richness of the family in the Western Palaearctic, and is considerably higher than the whole Caucasus (three species in two genera), adjacent Turkey (two species in two genera) and all of Europe (six species in two genera). Although this indicates a high diversity of this group in Iran, an even higher diversity should be expected, considering that most regions of Iran, especially the large Zagros Mountain range in the western parts, have never been thoroughly studied in regards to the filistatid fauna. We expect the occurrence of at least three additional genera in Iran: *Microfilistata* Zonstein, 1990, *Pritha* Lehtinen, 1967 and *Tricalamus* Wang, 1987. All these genera are known in adjacent Afghanistan, Azerbaijan and Turkmenistan (Zonstein et al. 2013, Mikhailov 2013).

## Supplementary Material

XML Treatment for Filistata

XML Treatment for Filistata insidiatrix

XML Treatment for Filistata lehtineni

XML Treatment for Sahastata

XML Treatment for Sahastata sinuspersica

XML Treatment for Zaitunia

XML Treatment for Zaitunia akhanii

XML Treatment for Zaitunia alexandri

XML Treatment for Zaitunia medica

XML Treatment for Zaitunia persica
